# Identification of a cytosine methyltransferase that improves transformation efficiency in *Methylomonas* sp. DH-1

**DOI:** 10.1186/s13068-020-01846-1

**Published:** 2020-12-07

**Authors:** Jun Ren, Hyang-Mi Lee, Thi Duc Thai, Dokyun Na

**Affiliations:** Department of Biomedical Engineering, Chung-Ang University, Seoul, 06974 Republic of Korea

**Keywords:** *Methylomonas* sp. DH-1, Transformation efficiency, DNA methylation, Cytosine methyltransferase

## Abstract

**Background:**

Industrial biofuels and other value-added products can be produced from metabolically engineered microorganisms. *Methylomonas* sp. DH-1 is a candidate platform for bioconversion that uses methane as a carbon source. Although several genetic engineering techniques have been developed to work with *Methylomonas* sp. DH-1, the genetic manipulation of plasmids remains difficult because of the restriction-modification (RM) system present in the bacteria. Therefore, the RM system in *Methylomonas* sp. DH-1 must be identified to improve the genetic engineering prospects of this microorganism.

**Results:**

We identified a DNA methylation site, TGGCCA, and its corresponding cytosine methyltransferase for the first time in *Methylomonas* sp. DH-1 through whole-genome bisulfite sequencing. The methyltransferase was confirmed to methylate the fourth nucleotide of TGGCCA. In general, methylated plasmids exhibited better transformation efficiency under the protection of the RM system than non-methylated plasmids did. As expected, when we transformed *Methylomonas* sp. DH-1 with plasmid DNA harboring the *psy* gene, the metabolic flux towards carotenoid increased. The methyltransferase-treated plasmid exhibited an increase in transformation efficiency of 2.5 × 10^3^ CFU/μg (124%). The introduced gene increased the production of carotenoid by 26%. In addition, the methyltransferase-treated plasmid harboring anti-*psy* sRNA gene exhibited an increase in transformation efficiency by 70% as well. The production of carotenoid was decreased by 40% when the *psy* gene was translationally repressed by anti-*psy* sRNA.

**Conclusions:**

Plasmid DNA methylated by the discovered cytosine methyltransferase from *Methylomonas* sp. DH-1 had a higher transformation efficiency than non-treated plasmid DNA. The RM system identified in this study may facilitate the plasmid-based genetic manipulation of methanotrophs.

## Background

Although methane contributes to the greenhouse effect much more than carbon dioxide does, it is a useful feedstock for methanotrophs, which are bacteria that utilize methane as a carbon source [1–3]. Methane can be converted into methanol [4], and methanol can be metabolized to many other value-added chemicals such as l-glutamate [5, 6], l-lysine [7, 8], cadaverine [9, 10], α-humulene [11], mesaconate, and (2S)-methyl-succinate [12] in metabolically engineered methanotrophs [13].

For efficient genetic engineering, genetic manipulation tools have been developed to work with methanotrophs [14–17]. Recently, the type l *Methylomonas* sp. DH-1 was isolated from brewery waste sludge, and several engineering tools have been developed [18]. This bacterium has been favored in diverse examples of metabolic engineering: the conversion of methane to methanol [18] and the production of value-added chemicals such as acetone [19, 20], succinate [21], and d-lactate [22].

The first hurdle in genetic engineering is to develop an efficient transformation method. In prokaryotes, DNA methylation and degradation by restriction-modification (RM) systems, which are rudimentary bacterial immune systems, are yet to be identified [23]. Usually, foreign DNA is not methylated and is thus destroyed by host restriction enzymes. The methylation of particular sequences in the host genome protects those sequences from cleavage by host restriction enzymes [24, 25]. A previous study showed that 88% of bacterial genomes contain RM systems and that 44% of bacterial genomes carry four or more RM systems [26]. Recently, the process of DNA methylation is utilized for epigenetic regulation [27] and nanopore sequencing [28].

Although several genetic manipulation techniques have been developed to metabolically engineer *Methylomonas* sp. DH-1 [29], the low transformation efficiency due to the inherent RM system has been an obstacle. In this study, we aimed to identify the RM system in *Methylomonas* sp. DH-1 and use it for enhanced genetic manipulation with plasmid DNA. Discovering the RM system of *Methylomonas* sp. DH-1 would enable the establishment of transformation techniques for efficient genetic manipulation.

## Results and discussion

### Identification of *Methylomonas* sp. DH-1 methylation site

**Fig. 1:**
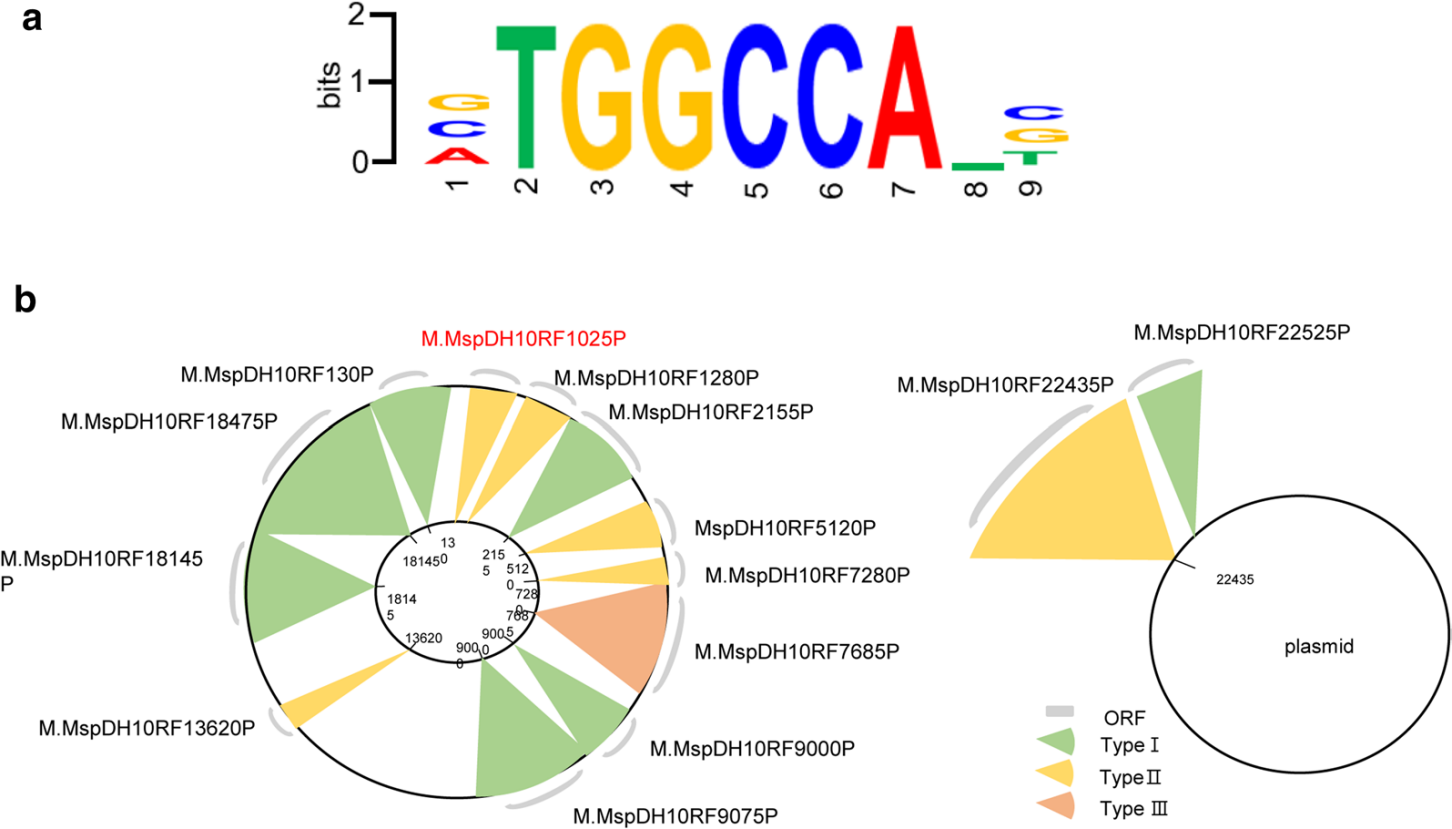
Identification of a methylation site and its corresponding methyltransferase in *Methylomonas* sp. DH-1. **a** The TGGCCA methylation site was the only site discovered by WGBS. **b** The potential methyltransferases in *Methylomonas* sp. DH-1. The REBASE-predicted methyltransferases in its genome and native plasmid are shown

To identify the RM system, the genome of *Methylomonas* sp. DH-1 was analyzed by whole-genome bisulfite sequencing (WGBS). Interestingly, only the TGGCCA motif was identified (Fig. 1a). In the REBASE database [30, 31], *Methylomonas* sp. DH-1 contains 12 RM systems in its genome and two in its native plasmid (Fig. 1b). According to REBASE, it was predicted that the cytosine methyltransferase AYM39_01025 (Additional file 1) would recognize the GGCC sequence for methylation, which is similar to the identified methylation site TGGCCA, in which the fourth nucleotide (C) was methylated in our results. Therefore, this cytosine methyltransferase was selected as a potential methylase for TGGCCA.

### Digestion protection assay

To investigate whether the selected cytosine methyltransferase (AYM39_01025) was able to recognize the identified sequence (TGGCCA) instead of the predicted sequence (GGCC), we conducted a DNA protection assay against digestion, using several restriction enzymes. When the cytosine methyltransferase protein was over-expressed in *E. coli* BL21 (DE3), the protein formed an inclusion body even though it was co-expressed with chaperones (pGro7 and pTf16). Thus, we could not perform the in vitro assay requiring purified methyltransferase. Instead, we introduced a plasmid harboring the methyltransferase gene and TGGCCA sites into the *E. coli* JM110 strain (*dam* and *dcm* methylase genes were deleted). Since the cytosine methyltransferase was under the control of the T5 promoter with a *lac* operator, we could obtain a non-methylated or methylated plasmid by IPTG. For further analysis, the plasmid was extracted from *E. coli* JM110 strain.

**Fig. 2:**
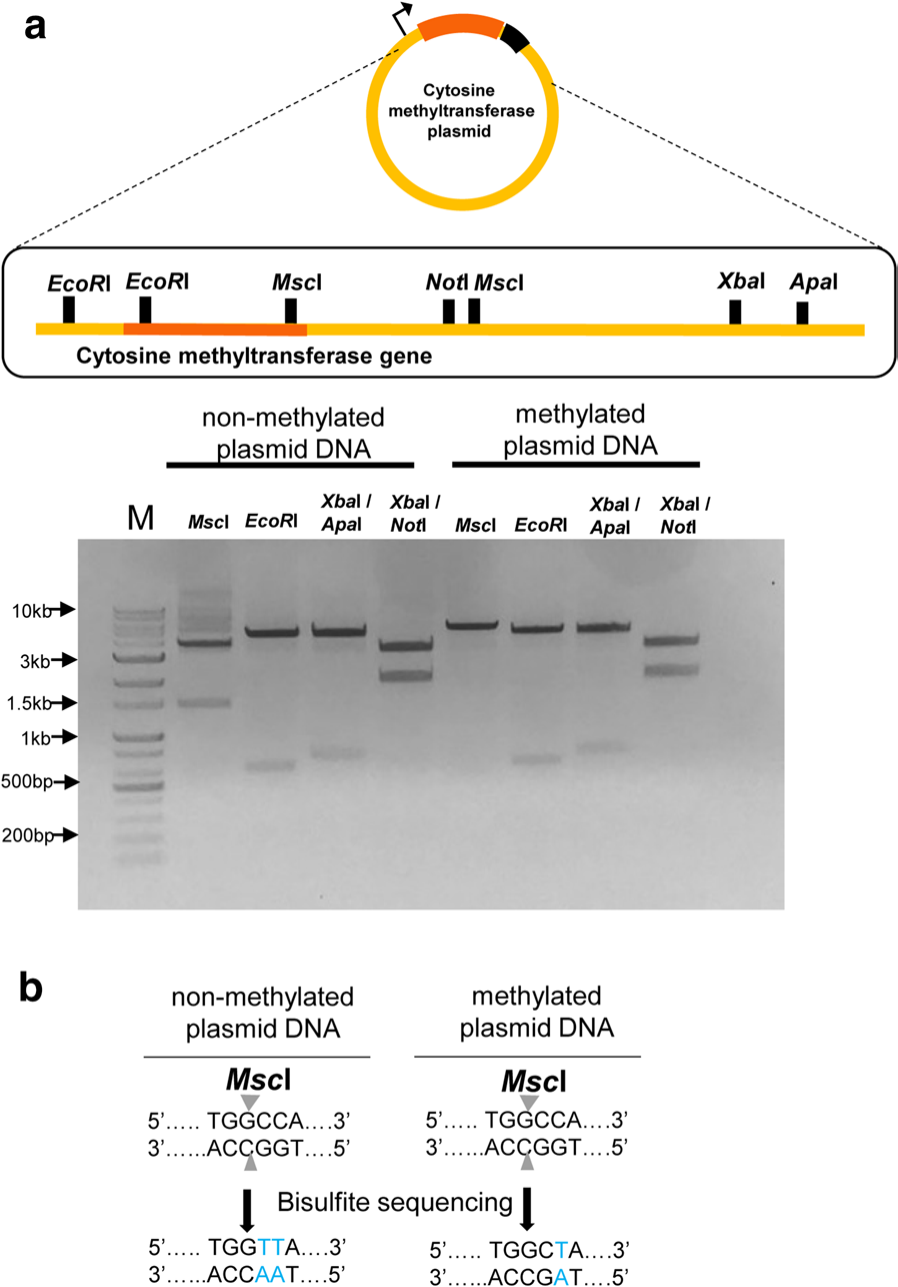
Agarose gel electrophoresis of the plasmid DNA cleaved by restriction enzymes. **a** Restriction patterns of non-methylated and methylated plasmid DNA. The plasmid map and restriction sites are also shown. Methylation was induced by 0.1 mM IPTG, which initiates the expression of the cytosine methyltransferase gene. *M* denotes a DNA marker. The treated restriction enzymes are shown in each lane. **b** The conversion of non-methylated and methylated TGGCCA sequences during bisulfite sequencing. Only non-methylated cytosines are converted to thymines during bisulfite sequencing and PCR. The left-hand figure shows that the two cytosines were converted to uracils, which means there were no methylated cytosines. Conversely, the right-hand figure shows that the fourth cytosine was not changed to uracil, indicating that it was methylated by the cytosine methyltransferase

According to the REBASE annotations, the cytosine methyltransferase of *Methylomonas* sp. DH-1 was predicted to methylate the GGCC sequence, while the only methylation site identified in *Methylomonas* sp. DH-1 by WGBS was TGGCCA. To confirm that the cytosine methyltransferase recognized TGGCCA instead of GGCC, several restriction enzymes that contain GGCC in their restriction sites were used: *Msc*I (TGGCCA), *Apa*I (GGGCCC), and *Not*I (GCGGCCGC). We also used *EcoR*l (GAATTC) and *Xba*l (TCTAGA) restriction enzymes as negative controls. The plasmid harboring the cytosine methyltransferase gene contained all of the above-mentioned restriction sites, as well. If the methylation site was GGCC, the restriction enzymes (*Msc*I, *Apa*I, and *Not*I) would not be able to cleave the plasmid DNA. As shown in Fig. 2a, most restriction enzymes were able to cleave both the non-methylated and methylated plasmids, but *Msc*I failed to cleave the methylated plasmid. This result indicated that the cytosine methyltransferase recognized TGGCCA and not GGCC.

To identify the cytosine nucleotide methylated by the cytosine methyltransferase, the methylated plasmid was analyzed by bisulfite sequencing. In bisulfite sequencing, only non-methylated cytosines are converted to uracil, and during PCR, the uracil is converted to T. Methylated cytosines are not changed by bisulfite sequencing. As shown in Fig. 2b, TGGCCA in the non-methylated plasmid was converted to TGGTTA, indicating that the cytosines were non-methylated, as expected. In the methylated plasmid, only the fifth cytosine in TGGCCA was converted to T, indicating that the fourth cytosine was methylated by the cytosine methyltransferase.

### Methylation of plasmid DNA increased transformation efficiency

**Fig. 3:**
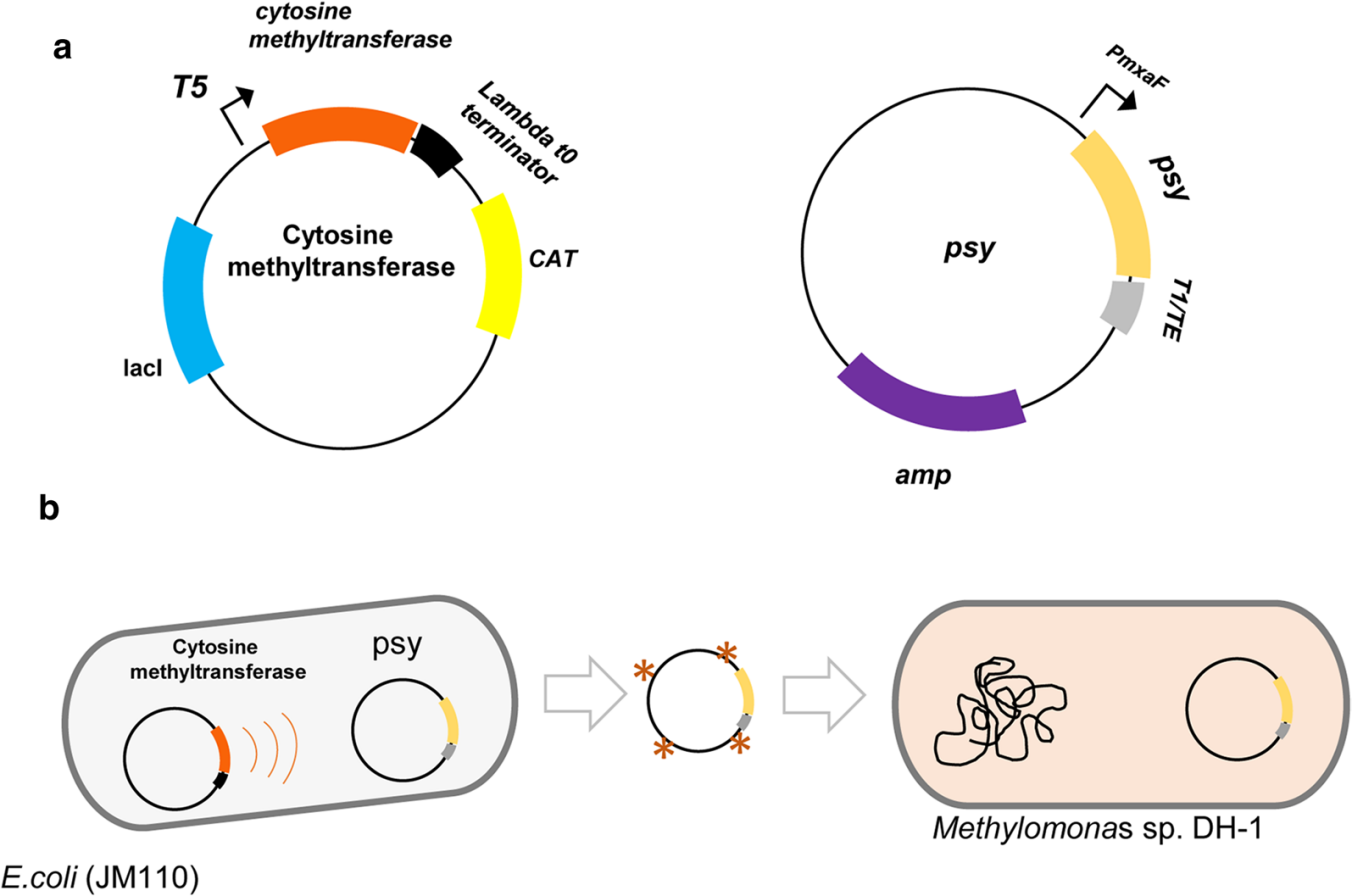
Plasmid maps of the constructed plasmids. **a** The cytosine methyltransferase-containing plasmid and *psy*-containing plasmid are shown. The *psy-*containing plasmid was constructed in a proof-of-concept metabolic engineering process to increase the metabolic flux towards carotenoid, and the cytosine methyltransferase-containing plasmid was used to methylate the *psy-*containing plasmid. The *psy* gene was under the control of the *mxaF* promoter, encoding subunit of methanol dehydrogenase [32], which was predicted using Promoter Hunter [37]. **b** The overall strategy of plasmid methylation in *E. coli* (JM110) and transformation into *Methylomonas* sp. DH-1. The first step was to methylate the target plasmid using cytosine methyltransferase, and the second step was to transform it into *Methylomonas* sp. DH-1 after separation from the methylase-containing plasmid

The plasmid harboring the *psy* (phytoene synthase) gene was constructed (Fig. 3a and Additional file 2) and co-transformed into *E. coli* JM110 with the plasmid harboring the cytosine methyltransferase gene. The *psy* gene is involved in the biosynthetic pathway that produces carotenoids. For the methylation of the plasmid containing *psy*, the media were supplemented with 0.1 mM IPTG to induce the expression of the cytosine methyltransferase. Since *E. coli* contains two plasmids (*psy* plasmid + cytosine methyltransferase plasmid), the plasmids were separated by gel electrophoresis, and the *psy* plasmid was extracted from the gel (Fig. 3b). The non-methylated plasmid was also extracted from the cell without IPTG to create a control sample in which the expression of the cytosine methyltransferase was not induced.

**Fig. 4:**
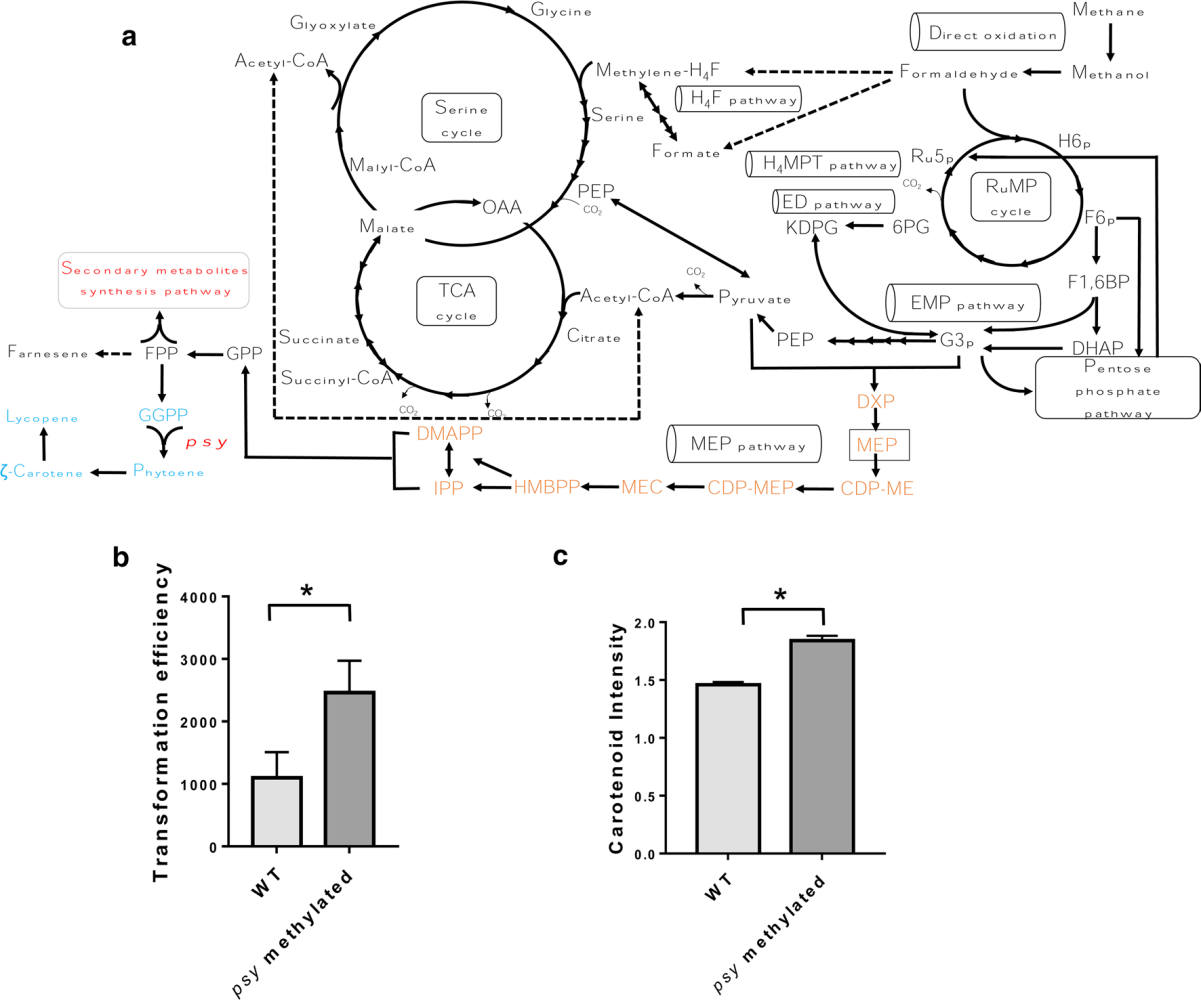
The transformation efficiency of methylated plasmid DNAs. **a** The overall biosynthetic pathway towards carotenoid. The *psy* gene is indicated. **b** Transformation efficiencies of non-methylated plasmids (light gray bar) and methylated plasmids (dark gray bar) in *Methylomonas* sp. DH-1. The maps of the two plasmids are shown in Fig. 3a. Standard deviations were calculated from triplicates. The asterisk (*) denotes *p* values < 0.05. **c** Carotenoid intensity in *Methylomonas* sp. DH-1 cells after transformation with the methylated *psy* plasmid. The intensity was measured using multi-detection microplate reader, and the carotenoid intensity was obtained 8 h after cultivation. Standard deviations were calculated from triplicates

The extracted plasmids were transformed into *Methylomonas* sp. DH-1 by electroporation. Since there are no artificial plasmids that exist separate from the genome of *Methylomonas* sp. DH-1, we measured the genome integration efficiencies of the *psy* gene involved in the carotenoid biosynthetic pathway (Fig. 4a) to deduce the transformation efficiency. The transformation efficiency of the methylated DNA of the *psy* plasmid was 2.5 × 10^3^ CFU/μg of DNA. The efficiency was increased by 124% compared with that of the non-methylated plasmid DNA (Fig. 4b). Despite of methylation, the efficiency increase was not dramatic. We think that the introduced plasmids were easily integrated into the genome of *Methylomonas* sp. DH-1 by recombinases and thus the protection of plasmids by methylation was not essential in genome integration experiments. However, to date there are no artificial plasmids available for *Methylomonas* sp. DH-1, which is independent from its genome. For the development of artificial plasmids, the protection of plasmids by methylation is essential and the identification of a cytosine methylation system is the first step for the development. In this regard, the newly identified methylation system would facilitate the development of artificial plasmids as well as other biotechnological techniques based on plasmids.

For further evaluation of methylation effect on transformation, we removed the three methylation sites in the *psy* plasmid by mutating nucleotides: one in an intergenic region and two in the coding region of *psy*. The former site was converted from TGGCCA to TGTCCA, and the latter two were converted from GTG GCC AAT to GTA GCG AAT and from CTG GCC AAA to CTA GCG AAA based on codon degeneracy not to mutate amino acids (Additional file 3: Fig S1a). As shown in Additional file 3: Fig. S1b, the deletion of the methylation sites increased transformation efficiency similar to that of the methylated *psy* plasmid. This indicates that methylation of plasmid DNA by the identified cytosine methyltransferase protects plasmid DNAs from the RM system of *Methylomonas* sp. DH-1 and increases the transformation efficiency, which may facilitate the genetic manipulation of *Methylomonas* sp. DH-1.

To investigate the effects of plasmid size and methylation on transformation efficiency, we constructed three different plasmids with a different length (5–7 kb). As shown in Additional file 3: Fig. S2, methylation increased transformation efficiency while plasmid length did not show any significant effect on transformation efficiency. This indicates that transformation efficiency is dependent on methylation, not plasmid size. Furthermore, we measured the growths of non-transformed wild-type cells and transformed cells with methylated plasmids (the *psy* plasmid or anti-*psy* sRNA plasmid). As shown in Additional file 3: Fig. S3, their growth rates are very similar, showing that there could be no significant changes in cellular physiology.

*Methylomonas* sp. DH-1 carries a complete MEP pathway for carotenoid production [19]. The selected gene, *psy*, is involved in the carotenoid biosynthetic pathway. The gene was designed to be expressed by the promoter of the *mxaF* gene [32] (Fig. 4a). When the plasmid containing the *psy* gene was introduced into *Methylomonas* sp. DH-1, the *psy* gene was integrated into the genome by homologous recombination. The additional copy of the *psy* gene increased carotenoid biosynthesis by 26% (Fig. 4c).

**Fig. 5:**
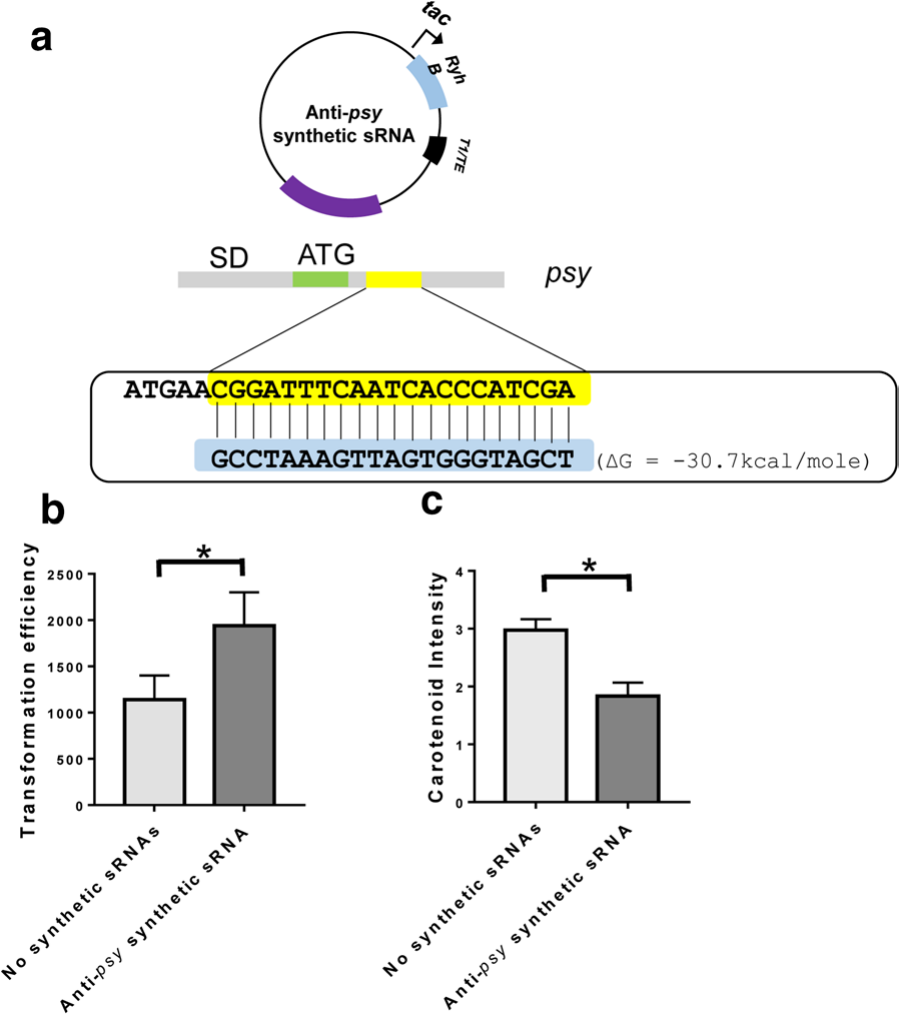
Transformation efficiency of a methylated plasmid harboring anti-*psy* synthetic sRNA gene. **a** An anti-*psy* synthetic sRNA gene was designed to reduce the expression of the *psy* gene. The synthetic sRNA binds to the coding region of the *psy* gene and represses the translation of the *psy* mRNA. The binding energy was calculated by *mfold* [38]. **b** Transformation efficiencies of the non-methylated plasmid (light gray bar) and methylated plasmid (dark gray bar) in *Methylomonas* sp. DH-1. Standard deviations were calculated from triplicates. The asterisk (*) denotes *p* values < 0.05. **c** Carotenoid intensity (arbitrary unit) in *Methylomonas* sp. DH-1 cells. The intensity was measured using multi-detection microplate reader, and the carotenoid intensity was obtained 8 h after cultivation. Standard deviations were calculated from triplicates. The asterisk (*) denotes *p* values < 0.05

Synthetic sRNAs have been utilized to increase the production of desired substances by regulating gene expression [33]. Synthetic sRNAs were designed to bind to the nucleotides in the translation initiation regions of mRNAs, and thereby they repressed the translation of mRNAs by preventing the binding of ribosomes in assistance of Hfq protein [34]. In this study, we constructed a plasmid containing an anti-*psy* synthetic sRNA gene to investigate the methylation effect on transformation efficiency and also to investigate the knock-down effect of the *psy* gene on carotenoid production (Fig. 5a and Additional file 4).

Synthetic sRNAs are composed of two elements: a target binding region and a scaffold. In previous studies, various scaffolds originated from *E. coli* were used including MicC and SgrS [33, 35]. Of many inherent sRNA genes, RyhB was known to operate in the absence of Hfq protein [36] and thus was expected to work in various bacterial species. We designed an anti-*psy* synthetic sRNA using the scaffold of RyhB sRNA. The anti-*psy* synthetic sRNA gene was under the control of *tac* promoter.

When the plasmid harboring the anti-*psy* synthetic sRNA gene was transformed after methylation, its transformation efficiency was enhanced by 70% compared with the non-methylated plasmid (Fig. 5b). In addition, when the synthetic sRNA gene was integrated into the genome of *Methylomonas* sp. DH-1, the synthetic sRNA decreased carotenoid production by 40% compared with that of wild-type *Methylomonas* sp. DH-1 (Fig. 5c). These results indicate that methylation of plasmids can improve transformation efficiency as well as that synthetic sRNAs based on a RyhB scaffold can be used to regulate the expression of genes in *Methylomonas* sp. DH-1.

## Conclusions

In this study, we identified a novel cytosine methyltransferase and its methylation site for the first time in *Methylomonas* sp. DH-1. The methylase was utilized to increase transformation efficiency by protecting plasmid DNAs from the RM system of *Methylomonas* sp. DH-1. Transformation is the first barrier in the genetic manipulation of bacteria, and with the aid of the methylase, the transformation barrier was effectively overcome. The use of the methylase for methylating insertional genes may facilitate the metabolic engineering of value-added products in *Methylomonas* sp. DH-1.

## Methods

### Strains, antibiotics, primers, and culture conditions

The *E. coli* DH5α strain was used for gene cloning and plasmid preparation, and the *E. coli* JM110 strain, *traD36 lacI*^*q*^Δ (*lacZ*) *M15 proA*^+^*B*^+^*IrpsL* (*Str*^*r*^) *thr leu thi lacY galK galT ara fhuA dam dcm glnV44 *Δ(*lac-proAB*) was used for plasmid methylation. *E. coli* cells were cultured in Luria–Bertani (LB) broth (1% tryptone, 0.5% yeast extract, and 1% NaCl) or on LB plates (1.5% w/v agar) at 37 °C in the presence of appropriate antibiotics (25 μg/mL of chloramphenicol and/or 10 μg/mL of Ampicillin). A cytosine methyltransferase (Additional file 1) expression vector was then constructed, and its expression was regulated by IPTG. For methylation, 0.1 mM IPTG was used. Next, the *psy* plasmid was constructed.

*Methylomonas* sp. DH-1 was cultured in a nitrate mineral salt (NMS) medium containing 10 μM CuCl_2_·2H_2_O as described previously [18]. *Methylomonas* sp. DH-1 cells were cultured in a 500 mL baffled flask sealed with a screw cap containing 100 mL of NMS medium at 30 °C and 250 rpm. Methane was supplied to a final concentration of 30% (v/v) by gas substitution using a gas-tight syringe, and the headspace was refreshed daily. During carotenoid intensity measurement, methanol (0.1%) was used as a carbon source instead of methane because the *Methylomonas* sp. DH-1 cells were cultured in a 96-well plate.

### Whole-genome bisulfite sequencing and plasmid construction

A whole-genome bisulfite sequencing of *Methylomonas* sp. DH-1 was carried out according to the manufacturer’s instruction [39]. Briefly, genomic DNA of *Methylomonas* sp. DH-1 was extracted and was treated with bisulfite to convert unmethylated cytosines to uracils while retaining those which were methylated. Bisulfite-treated ssDNA fragments were randomly primed using a polymerase that can read uracil nucleotides to synthesize DNA strands containing a specific sequence tag. The 3′ ends of the newly synthesized DNA strands were then selectively tagged with a second sequence tag. This process generated di-tagged DNA strands with known tags at their 5′ and 3′ ends. The di-tagged DNA strands were enriched by PCR, resulting in dsDNA strands.

After sequencing, the raw sequence reads were filtered based on quality. Trimming process was done to eliminate adapter sequences and bases with low quality from each read using Trimmomatic program [40]. The bases with low quality or N bases less than quality 3 from the ends of reads were trimmed. Also, using a sliding window method, bases of reads that did not qualify for a 4-base wide sliding window, and the average quality per window below 15 were trimmed. Afterwards, reads with length shorter than 36 bp were dropped to produce cleaned data. The trimmed reads were mapped to a reference genome (*Methylomonas* sp. DH-1, ASM164468v1) with BSMAP based on SOAP (Short Oligo Alignment Program), which is a short reads mapping program for bisulfite sequencing in DNA methylation study [41]. The nucleotides around methylated cytosines (− 10 ~ + 10 nt) were extracted and consensus sequence motifs were identified using MEME [42]. As a result, one single motif was identified (TGGCCA). The raw data files obtained from bisulfite sequencing are available to download at http://ssbio.cau.ac.kr/public/DH-1_1.fastq.gz and http://ssbio.cau.ac.kr/public/DH-1_2.fastq.gz.

### *Methylomonas* sp. DH-1 electroporation

*Methylomonas* sp. DH-1 was grown in a nitrate mineral salt (NMS) plate containing 10 μM CuCl_2_·2H_2_O as described previously [23]. Cells were collected from plates using a spreader and resuspended in distilled water (DW) to make OD_600_ = 0.8. 10 mL of the resuspended cells were harvested by centrifugation at 5000 rpm at 4 °C for 10 min. The pellet was washed with 10 mL of DW, transferred to a 15 mL conical tube, and centrifuged again at 5000 rpm at 4 °C for 10 min. The resulting pellet was resuspended in 100 μL of DW and placed on ice. Fifty microliters of the resuspended cells were gently mixed with plasmid 500 ng of DNA (3–5 μL), and the mixture was transferred to an ice-cold 1 mm micropulser electroporation cuvettes (Bio-Rad, Hercules, California 94547, United States). Electroporation was performed using a micropulser electroporator (Gene Pulser II System, Bio-Rad, Herules, California 94547, United States) at 25 μF and 200 Ω. After electrical discharge, 1 mL of NMS medium was immediately added to the cells. The cells were transferred into a 250 mL serum bottle with 10 mL of NMS medium and then incubated with 0.02% methane gas. After incubation at 30 °C for 4 h, the cells were collected by centrifugation at 5000 rpm for 10 min at 25 °C. The cells were resuspended with 1 mL of NMS medium and spread onto selective NMS plates.

### Carotenoid measurement

*Methylomonas* sp. DH-1 transformed with the *psy* plasmid (Additional file 2) was cultured in NMS medium containing methanol (0.1%) at 30 °C until the stationary phase was reached. The *Methylomonas* sp. DH-1 was transferred to a 96-well plate containing 200 μL of NMS medium and grown in a shaking format at 30 °C. The OD_450_ absorbance at 8 h was measured to infer the relative carotenoid concentration using a multi-detection microplate reader (SpectraMax M2, Molecular Devices, Sunnyvale, CA, USA).

## Supplementary Information

**Additional file 1.** Cytosine methyltransferase gene sequence in SnapGene file format.

**Additional file 2.** Psy plasmid map in SnapGene file format.

**Additional file 3: Fig. S1.** Transformation efficiency of the plasmid DNA in which methylation sites were changed to non-methylation sites. **Fig. S2.** The effects of plasmid length and methylation on transformation efficiency. **Fig. S3.** The growth curves of *Methylomonas *sp. DH-1 cells.

**Additional file 4.** Anti-psy sRNA plasmid map in SnapGene file format.

## Data Availability

Not applicable.

## Abbreviations

RM: Restriction modification
*psy*: Phytoene synthase
NMS: Nitrate mineral salt
DW: Distilled water
WGBS: Whole-genome bisulfite sequencing
IPTG: Isopropyl β-d-1-thiogalactopyranoside
sRNA: Regulatory small RNA
TIR: Translation initiation region

## References

[1] Clomburg JM, Crumbley AM, Gonzalez R (2017). Industrial biomanufacturing: the future of chemical production. Science.

[2] Hwang IY, Hur DH, Lee JH, Park CH, Chang IS, Lee JW (2015). Batch conversion of methane to methanol using *Methylosinus trichosporium* OB3b as biocatalyst. J Microbiol Biotechnol.

[3] Kalyuzhnaya MG, Puri AW, Lidstrom ME (2015). Metabolic engineering in methanotrophic bacteria. Metab Eng.

[4] Xin JY, Cui JR, Niu JZ, Hua SF, Xia CG, Li SB (2004). Production of methanol from methane by methanotrophic bacteria. Biocatal Biotransform.

[5] Brautaset T, Jakobsen OM, Degnes KF, Netzer R, Naerdal I, Krog A (2010). *Bacillus methanolicus* pyruvate carboxylase and homoserine dehydrogenase I and II and their roles for l-lysine production from methanol at 50 degrees C. Appl Microbiol Biotechnol.

[6] Brautaset T, Williams MD, Dillingham RD, Kaufmann C, Bennaars A, Crabbe E (2003). Role of the *Bacillus methanolicus* citrate synthase II gene, citY, in regulating the secretion of glutamate in l-lysine-secreting mutants. Appl Environ Microbiol.

[7] Naerdal I, Netzer R, Ellingsen TE, Brautaset T (2011). Analysis and manipulation of aspartate pathway genes for l-lysine overproduction from methanol by *Bacillus methanolicus*. Appl Environ Microbiol.

[8] Jakobsen OM, Brautaset T, Degnes KF, Heggeset TM, Balzer S, Flickinger MC (2009). Overexpression of wild-type aspartokinase increases l-lysine production in the thermotolerant methylotrophic bacterium *Bacillus methanolicus*. Appl Environ Microbiol.

[9] Naerdal I, Pfeifenschneider J, Brautaset T, Wendisch VF (2015). Methanol-based cadaverine production by genetically engineered *Bacillus methanolicus* strains. Microb Biotechnol.

[10] Irla M, Heggeset TM, Naerdal I, Paul L, Haugen T, Le SB (2016). Genome-based genetic tool development for *Bacillus methanolicus*: theta- and rolling circle-replicating plasmids for Inducible gene expression and application to methanol-based cadaverine production. Front Microbiol.

[11] Sonntag F, Kroner C, Lubuta P, Peyraud R, Horst A, Buchhaupt M (2015). Engineering *Methylobacterium extorquens* for de novo synthesis of the sesquiterpenoid alpha-humulene from methanol. Metab Eng.

[12] Sonntag F, Buchhaupt M, Schrader J (2014). Thioesterases for ethylmalonyl-CoA pathway derived dicarboxylic acid production in *Methylobacterium extorquens* AM1. Appl Microbiol Biotechnol.

[13] Pfeifenschneider J, Brautaset T, Wendisch VF (2017). Methanol as carbon substrate in the bio-economy: metabolic engineering of aerobic methylotrophic bacteria for production of value-added chemicals. Biofuel Bioprod Biorefin.

[14] Puri AW, Owen S, Chu F, Chavkin T, Beck DA, Kalyuzhnaya MG (2015). Genetic tools for the industrially promising methanotroph *Methylomicrobium buryatense*. Appl Environ Microbiol.

[15] Yan X, Chu F, Puri AW, Fu Y, Lidstrom ME (2016). Electroporation-based genetic manipulation in type I methanotrophs. Appl Environ Microbiol.

[16] Crombie A, Murrell JC. Development of a system for genetic manipulation of the facultative methanotroph *Methylocella silvestris* Bl2. In: Methods in enzymology. 2011. pp. 119–33. 10.1016/B978-0-12-386905-0.00008-5 21419918

[17] Ojala DS, Beck DAC, Kalyuzhnaya MG. Genetic systems for moderately halo (alkali)philic bacteria of the genus methylomicrobium. In: Methods in enzymology. 2011. pp. 99–118. 10.1016/B978-0-12-386905-0.00007-3 21419917

[18] Hur DH, Na JG, Lee EY (2017). Highly efficient bioconversion of methane to methanol using a novel type I *Methylomonas* sp DH-1 newly isolated from brewery waste sludge. J Chem Technol Biotechol.

[19] Nguyen AD, Hwang IY, Lee OK, Hur DH, Jeon YC, Hadiyati S (2018). Functional analysis of *Methylomonas* sp DH-1 genome as a promising biocatalyst for bioconversion of methane to valuable chemicals. Catalysts.

[20] Hur DH, Nguyen TT, Kim D, Lee EY (2017). Selective bio-oxidation of propane to acetone using methane-oxidizing *Methylomonas* sp. DH-1. J Ind Microbiol Biotechnol.

[21] Nguyen DTN, Lee OK, Hadiyati S, Affifah AN, Kim MS, Lee EY (2019). Metabolic engineering of the type I methanotroph *Methylomonas* sp. DH-1 for production of succinate from methane. Metab Eng.

[22] Lee JK, Kim S, Kim W, Kim S, Cha S, Moon H (2019). Efficient production of d-lactate from methane in a lactate-tolerant strain of *Methylomonas* sp. DH-1 generated by adaptive laboratory evolution. Biotechnol Biofuels.

[23] Bertani G, Weigle JJ (1953). Host controlled variation in bacterial viruses. J Bacteriol.

[24] Murray NE (2000). Type I restriction systems: sophisticated molecular machines (a legacy of Bertani and Weigle). Microbiol Mol Biol Rev.

[25] Hornby DP (1993). DNA methyltransferases (EC 2.1.1.72 and EC 2.1.1.73). Methods Mol Biol.

[26] Roberts RJ, Vincze T, Posfai J, Macelis D (2007). REBASE—enzymes and genes for DNA restriction and modification. Nucleic Acids Res.

[27] Oliveira PH, Fang G. Conserved DNA methyltransferases: a window into fundamental mechanisms of epigenetic regulation in bacteria. Trends Microbiol. 2020. 10.1016/j.tim.2020.04.007 PMC7666040 32417228

[28] Tourancheau A, Mead EA, Zhang X-S, Fang GJb. Discovering and exploiting multiple types of DNA methylation from individual bacteria and microbiome using nanopore sequencing. 2020. 10.1038/s41592-021-01109-3 PMC8107137 33820988

[29] Ren J, Na D, Yoo SM (2018). Optimization of chemico-physical transformation methods for various bacterial species using diverse chemical compounds and nanomaterials. J Biotechnol.

[30] Roberts RJ, Vincze T, Posfai J, Macelis D (2003). REBASE: restriction enzymes and methyltransferases. Nucleic Acids Res.

[31] Roberts RJ, Vincze T, Posfai J, Macelis D (2005). REBASE—restriction enzymes and DNA methyltransferases. Nucleic Acids Res.

[32] Chistoserdova L, Kuhn M, Lidstrom ME (1994). Identification of a promoter region for *mxaF* (*moxF*) from the type I methanotroph, *Methylobacter albus* BG8. FEMS Microbiol Lett.

[33] Na D, Yoo SM, Chung H, Park H, Park JH, Lee SY (2013). Metabolic engineering of *Escherichia coli* using synthetic small regulatory RNAs. Nat Biotechnol.

[34] De Lay N, Schu DJ, Gottesman S (2013). Bacterial small RNA-based negative regulation: Hfq and its accomplices. J Biol Chem.

[35] Noh M, Yoo SM, Yang D, Lee SY (2019). Broad-spectrum gene repression using scaffold engineering of synthetic sRNAs. Acs Synth Biol.

[36] Hao Y, Zhang ZJ, Erickson DW, Huang M, Huang Y, Li J (2011). Quantifying the sequence-function relation in gene silencing by bacterial small RNAs. Proc Natl Acad Sci USA.

[37] Klucar L, Stano M, Hajduk M (2010). phiSITE: database of gene regulation in bacteriophages. Nucleic Acids Res.

[38] Zuker M (2003). Mfold web server for nucleic acid folding and hybridization prediction. Nucleic Acids Res.

[39] Nair SS, Luu PL, Qu WJ, Maddugoda M, Huschtscha L, Reddel R (2018). Guidelines for whole genome bisulphite sequencing of intact and FFPET DNA on the Illumina HiSeq X Ten. Epigenet Chromatin.

[40] Bolger AM, Lohse M, Usadel B (2014). Trimmomatic: a flexible trimmer for Illumina sequence data. Bioinformatics.

[41] Xi Y, Li W (2009). BSMAP: whole genome bisulfite sequence MAPping program. BMC Bioinform.

[42] Bailey TL, Boden M, Buske FA, Frith M, Grant CE, Clementi L (2009). MEME suite: tools for motif discovery and searching. Nucleic Acids Res.

