# Supplementary material for: Identification of a cytosine methyltransferase that improves transformation efficiency in Methylomonas sp. DH-1
Source: Biotechnol Biofuels. 2020 Dec 7;13:200. doi: 10.1186/s13068-020-01846-1 (PMC7720504; doi:10.1186/s13068-020-01846-1)
Supplement: Supplementary file 3 — Additional file 3: Fig. S1. Transformation efficiency of the plasmid DNA in which methylation sites were changed to non-methylation sites. Fig. S2. The effects of plasmid length and methylation on transformation efficiency. Fig. S3. The growth curves of Methylomonas sp. DH-1 cells. [file 13068_2020_1846_MOESM3_ESM.docx]

**Additional information**

**Identification of a cytosine methyltransferase that improves transformation efficiency in *Methylomonas* sp.DH-1**

**Jun Ren, Hyang-Mi Lee, Thi Duc Thai, and Dokyun Na***

*Department of Biomedical Engineering, Chung-Ang University, Seoul 06974, Republic of Korea*

*Corresponding author:

Dokyun Na, PhD.

Associate Professor

E-mail addresses*:* blisszen@cau.ac.kr


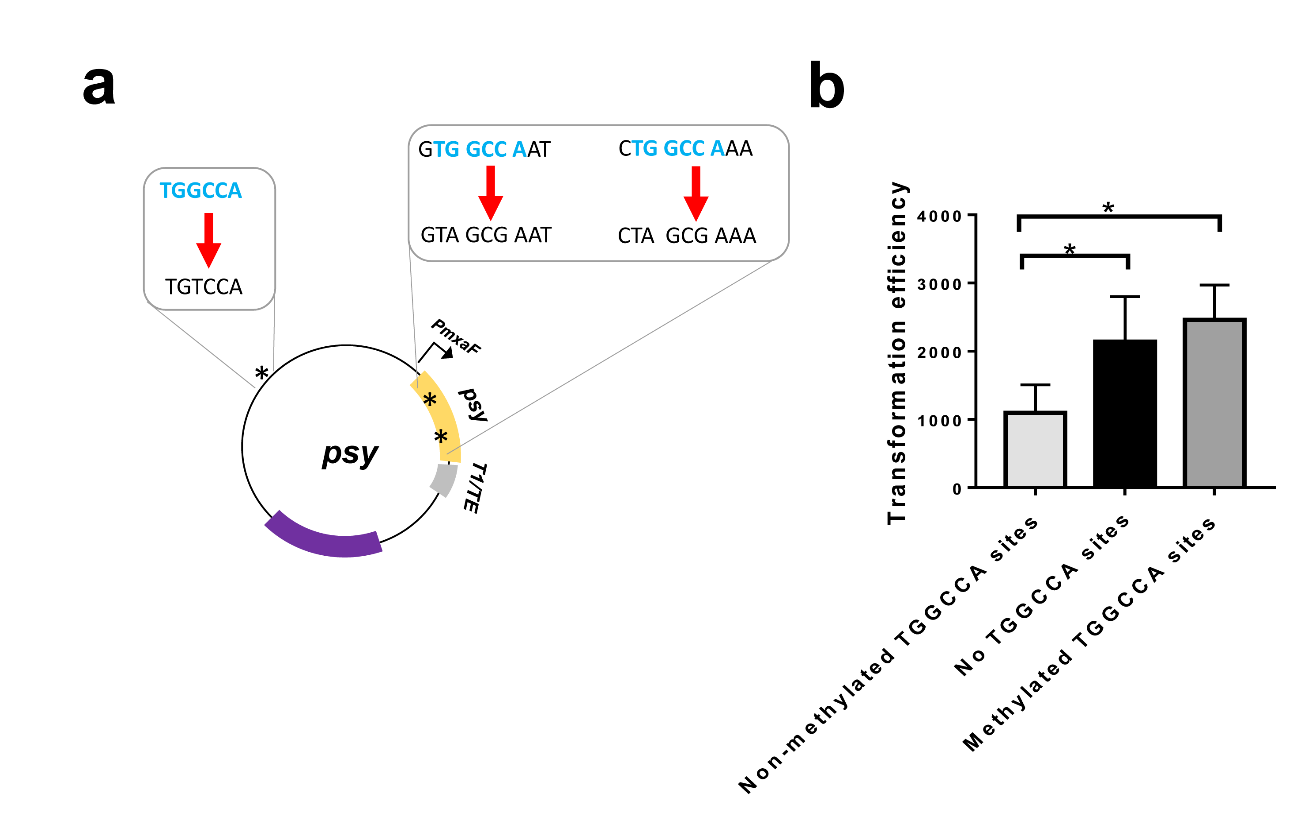


**Fig. S1.** Transformation efficiency of the plasmid DNA in which methylation sites were changed to non-methylation sites.

(a) The *psy* plasmid map and methylation sites are shown. The two methylation sites in the coding region of the *psy* gene were changed using codon degeneracy in order not to change amino acids. (b) Three methylation sites in the *psy* plasmid were changed to non-methylation sites to investigate the methylation effect on transformation efficiency. Light gray bar, transformation efficiency of the non-methylated plasmid containing three methylation sites. Black bar, transformation efficiency of the non-methylated plasmid containing no methylation sites. Dark gray bar, transformation efficiency of the methylated plasmid containing methylation sites. Standard deviations were calculated from triplicates. The asterisk (*) denotes *p*-values < 0.05.

**
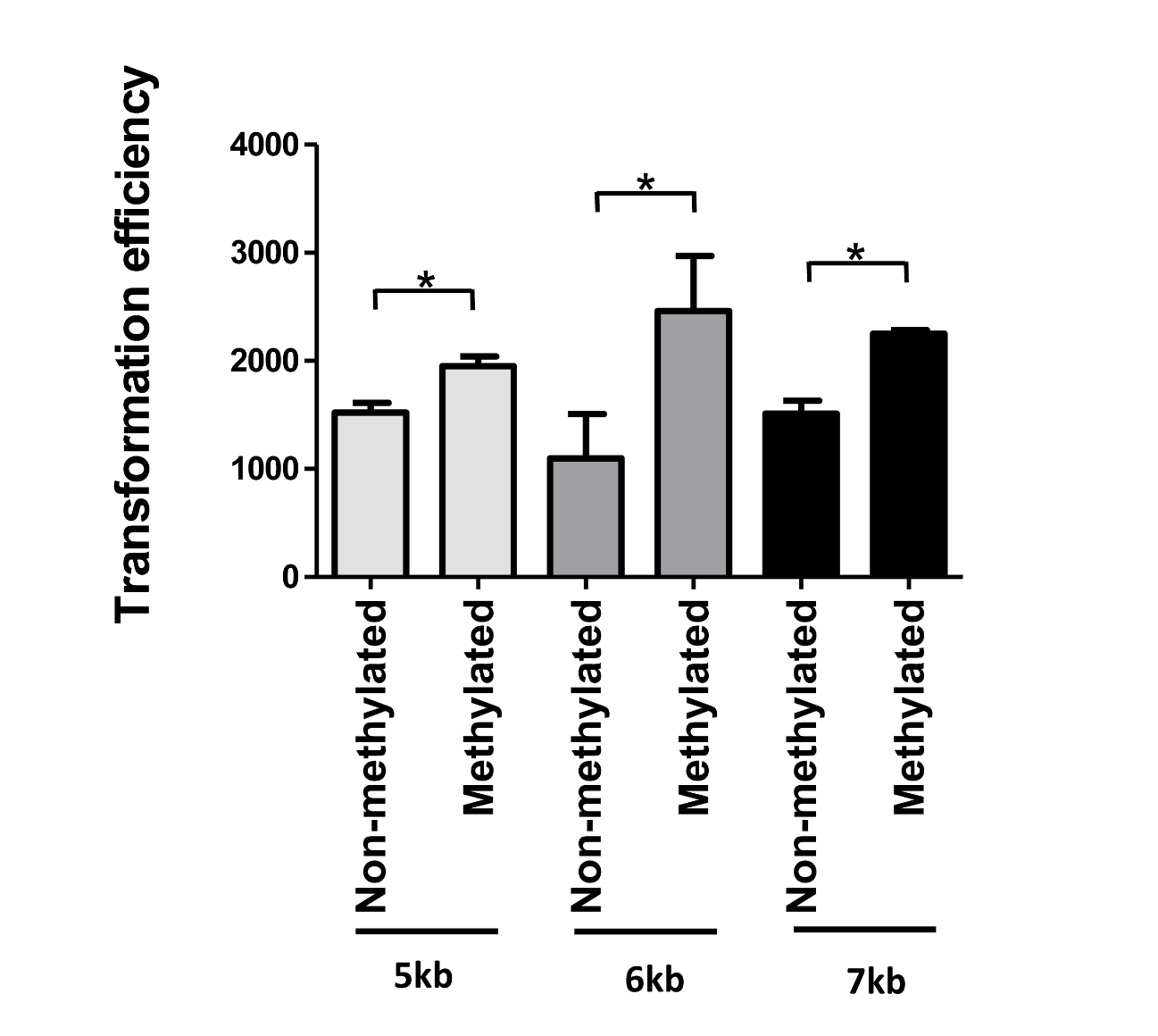
**

**Fig. S2.** The effects of plasmid length and methylation on transformation efficiency.

*Methylomonas* sp. DH-1 cells were transformation with methylated/non-methylated plasmids with various lengths. Standard deviations were calculated from triplicates. The asterisk (*) denotes *p*-values < 0.05

**
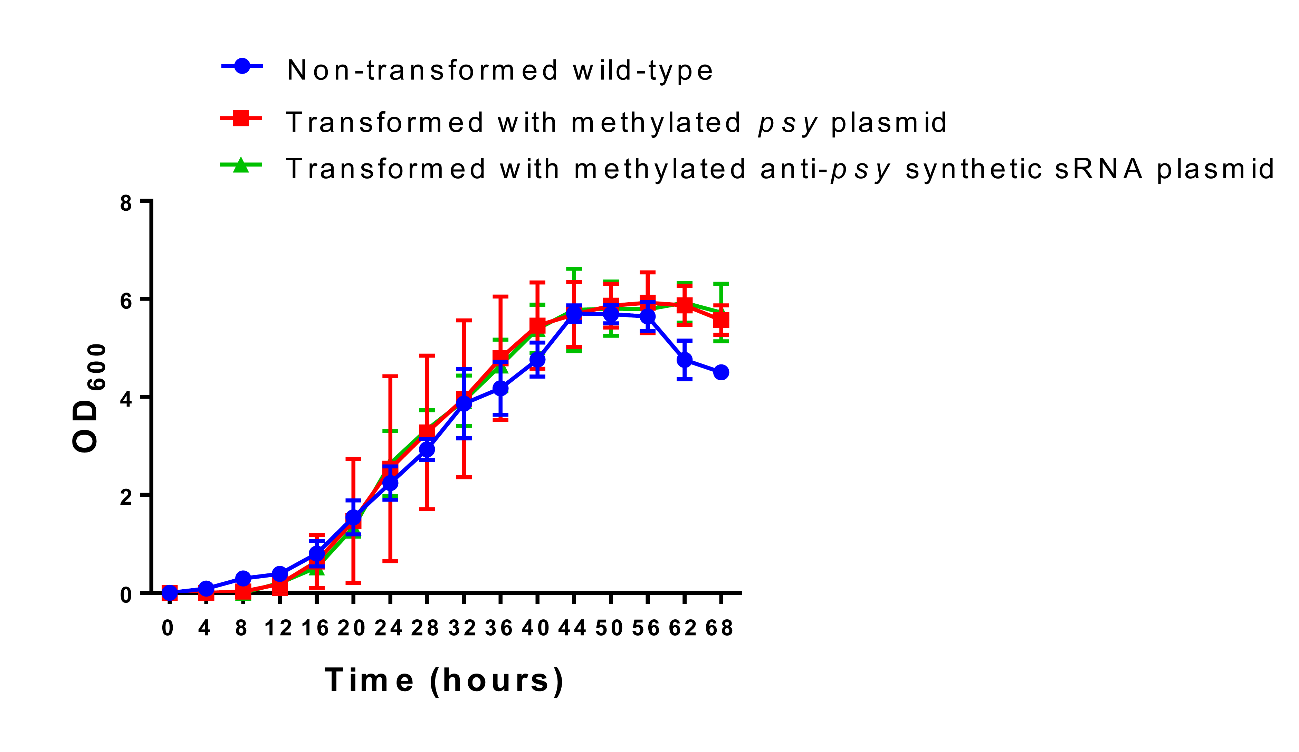
**

**Fig. S3.** The growth curves of *Methylomonas* sp. DH-1 cells.

The growth curves of non-transformed wild type cells (blue), cells transformed with the methylated psy plasmid (red), and cells transformed with the methylated anti-*psy* synthetic sRNA plasmid (green). Standard deviations were calculated from triplicates.
